# eNCApsulate: neural cellular automata for precision diagnosis on capsule endoscopes

**DOI:** 10.1007/s11548-025-03425-x

**Published:** 2025-07-04

**Authors:** Henry John Krumb, Anirban Mukhopadhyay

**Affiliations:** Computer Science Department, TU Darmstadt, Fraunhoferstr. 5, Darmstadt, 64293 Hessen Germany

**Keywords:** Wireless capsule endoscopy, Neural cellular automata, Edge AI, Image segmentation, Depth estimation

## Abstract

**Purpose:**

Wireless capsule endoscopy (WCE) is a noninvasive imaging method for the entire gastrointestinal tract and is a pain-free alternative to traditional endoscopy. It generates extensive video data that requires significant review time, and localizing the capsule after ingestion is a challenge. Techniques like bleeding detection and depth estimation can help with localization of pathologies, but deep learning models are typically too large to run directly on the capsule.

**Methods:**

Neural cellular automata (NCAs) for bleeding segmentation and depth estimation are trained on capsule endoscopic images. For monocular depth estimation, we distill a large foundation model into the lean NCA architecture, by treating the outputs of the foundation model as pseudo-ground truth. We then port the trained NCAs to the ESP32 microcontroller, enabling efficient image processing on hardware as small as a camera capsule.

**Results:**

NCAs are more accurate (Dice) than other portable segmentation models, while requiring more than 100x fewer parameters stored in memory than other small-scale models. The visual results of NCAs depth estimation look convincing and in some cases beat the realism and detail of the pseudo-ground truth. Runtime optimizations on the ESP32-S3 accelerate the average inference speed significantly, by more than factor 3.

**Conclusion:**

With several algorithmic adjustments and distillation, it is possible to eNCApsulate NCA models into microcontrollers that fit into wireless capsule endoscopes. This is the first work that enables reliable bleeding segmentation and depth estimation on a miniaturized device, paving the way for precise diagnosis combined with visual odometry as a means of precise localization of the capsule—on the capsule.

**Supplementary Information:**

The online version contains supplementary material available at 10.1007/s11548-025-03425-x.

## Introduction

Wireless capsule endoscopy (WCE) is a noninvasive imaging modality that allows to record the gastrointestinal (GI) tract in its entirety, including the otherwise hard to reach small intestine. This modality is compelling as it allows for pain-free and precise diagnosis, in a procedure that is not associated with stigma—unlike traditional endoscopy. However, capsule endoscopes collect hours of video data, which typically take 30 to 120 min of the physician’s time to review [1]. Video summarization and image classification are key technologies to counter this problem. Performing the classification on the capsule directly further helps to significantly reduce the amount of data to be transmitted, as in most patients, only a small fraction of images shows pathological findings.

Another issue is to localize the camera capsule after it was swallowed, which still poses a challenge to researchers and engineers. Knowing the exact location of the capsule allows for a targeted diagnosis and assists to locate the capsule in cases of retention, which is the most significant complication associated with WCE [2]. Several approaches discussed in the literature aim to include sophisticated sensors [3, 4] to accomplish this task, which require additional hardware to be built into the miniaturized capsule. Others propose to track a permanent magnet inside the capsule using magnetic sensor arrays on the outside of the patient [5], requiring the patient to wear a sensor array for more than 12 h while the capsule is traveling, which is uncomfortable and cumbersome.

Image-guided techniques like visual odometry (VO) are better suited for the task, as they enable to locate the capsule inside the GI tract only based on available image data [6]. One key ingredient to VO is depth images, which can be generated very well with contemporary monocular depth estimation models, even for a niche domain like WCE [6–8] which is underrepresented in training sets of foundation models. Even such monocular depth estimation models can be employed for VO, as long as they predict absolute depth maps. However, all VO approaches for WCE are analyzed on PC hardware due to their high demands to compute, utilizing retrospective data collections. As depth estimation models are too large for embedded platforms, precision navigation and diagnosis are not possible on the capsule.

Our vision is to bring diagnosis and visual odometry onto the capsule endoscope , enabling a localization of the capsule while it travels through the GI tract without the requirement for additional sensing. We start to pursue this vision by shrinking segmentation and depth estimation models to the size of a capsule-sized microcontroller , which would be impossible with contemporary convolutional neural network (CNN) or attention-based models. We employ NCAs models, which are a new family of bioinspired neural networks that are known to be not only accurate and robust, but also lightweight enough to fit on small-scale hardware at a minimal memory footprint [9].

**Fig. 1:**
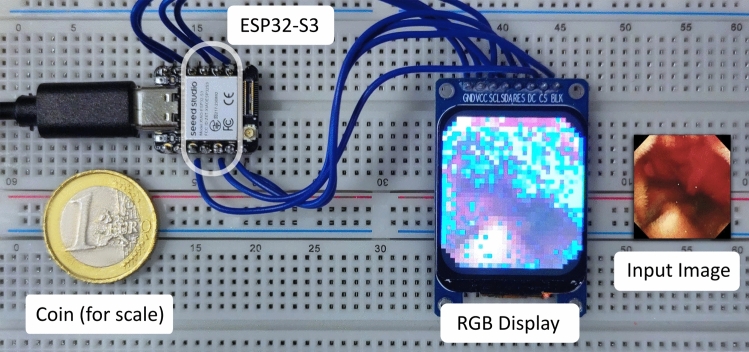
Experimental hardware setup for eNCApsulate, featuring a tiny version of the ESP32-S3 as the main component. The chip itself is miniaturized and can thus fit into a capsule endoscope (white contour overlay). An RGB display, connected directly to the chip, displays the segmentation of a capsule endoscopic image generated by an NCA

In this work, we present **eNCApsulate**, an approach for capsule endoscopic diagnosis and navigation that is lean enough to run on a capsule device itself. This miniaturization is achieved by leveraging NCA, an emerging lightweight and robust neural architecture, which we port to the ESP32 microcontroller. We find that NCA yield superior segmentations and convincing depth maps, while being 10x smaller than respective state-of-the-art models. We evaluate their performance on an unseen dataset, presenting quantitative and qualitative results.

Our contributions are threefold:

1. We are the first to introduce NCA models to the field of capsule endoscopy, showing that NCAs predict accurate and convincing bleeding segmentations. Our segmentation is $29.1\%$ more accurate (Dice) than the predictions of other small-scale segmentation models.
2. Our work is the first to distill a large foundation model into a small NCA. We investigate the task of depth estimation, finding that NCA produce convincing depth maps from RGB images by leveraging pseudo-ground truth from Depth Anything V2.
3. We demonstrate that our proposed approach can be ported to a microcontroller, such that the segmentation and depth estimation are carried out on the capsule itself. The distilled segmentation and depth estimation models are ported to an ESP32-S3 microcontroller, which is small enough to fit inside a common capsule endoscope. This is an initial step toward precise pathology diagnosis, combined with localization via VO on the capsule.

## Related work

**WCE Bleeding Detection:** The main research challenge in bleeding detection on WCE data is the scarcity of annotated data for this rather exotic modality. Even though available datasets promise a high quantity of images, they comprise mostly redundant healthy samples and only few bleeding images. The largest WCE dataset, KvasirCapsule [10], contains 47,238 annotated images, of which 446 show blood—these 446 images stem from only two individual patients. Vats et al. [11] address the issue of data scarcity by inducing domain priors into a contrastive learning scheme for bleeding detection, utilizing multiple datasets.

**WCE Depth Estimation:** With EndoSLAM, Ozyoruk et al. propose a holistic pipeline for depth estimation and VO for WCE, introducing a public dataset of multiple anatomies and capsules [6], in which depth maps were acquired in a simulated setting. Obtaining ground truth depth maps for monocular depth estimation in WCE is a challenge that is being actively investigated. In their recent study, Jeong et al. leverage simulated data to generate labeled ground truth and then use a CycleGAN-based approach to bridge the sim-to-real gap [12]. Universal depth estimators like the Depth Anything Model are also investigated [7, 8] for their applicability in endoscopic imaging and reconstruction. Although the more recent DepthAnythingV2 has not yet applied to WCE images, these studies show that there is an increased demand for monocular depth estimation on WCE images, and contemporary depth estimators achieve promising results.

**Miniaturized Models on Capsule Endoscopes:** Bringing advanced CNN-based models to the minimal capsule hardware is also a recent research challenge. Sahafi et al. pioneered this field by proposing a custom capsule design, featuring a Kendryte K210 chip, allowing to run simple CNN models on the capsule for the purpose of polyp classification [13]. However, simple CNN models do not scale equally for every downstream task and have a larger memory footprint for tasks like depth estimation or segmentation, prohibiting the deployment on the capsule.

**NCA on Minimal Hardware:** Portable architectures like NCAs promise to bring advanced neural image processing for universal downstream tasks to miniaturized hardware. For instance, Kalkhof et al. successfully ported NCAs to the Raspberry Pi [9, 14] and Smartphones [15]. In this work, we leverage NCAs to generate bleeding segmentation and depth images on hardware that is tiny enough to fit in a capsule endoscope. With our framework, we are able to generate convincing depth maps at a minimal memory footprint, which is otherwise impossible with contemporary CNN-type or transformer architectures.

## Methods

We start by explaining the general idea behind NCA training and inference and continue by outlining the eNCApsulate models with all of their tweaks. Finally, we will elaborate on the experimental design used in this study to train NCA models on the PC and transfer them to the ESP32 microcontroller platform Fig. 1.

### Neural cellular automata

**Fig. 2:**
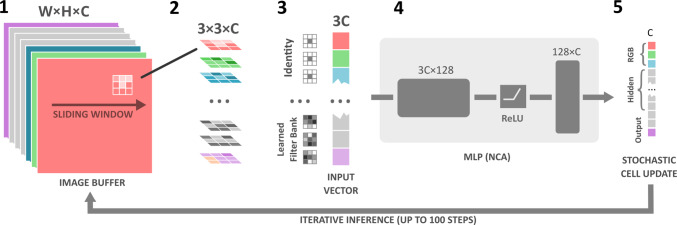
eNCApsulate architecture for lightweight segmentation or depth estimation. (1) The channels of the input RGB image are augmented to the match the input + hidden + output channel dimension *C*. (2) The input image is then processed by a learned bank of $3\times 3$ filters, and for each pixel, the concatenated result (3) is fed as an input to the NCA MLP network (4). The NCA MLP computes the image update for each cell. The result is an update vector (5) that is added to the input image buffer by a chance of 50% (stochastic cell update)

NCAs are an emerging family of neural models that are gaining traction in the application of medical image processing. Their working principle combines the ideas of two bioinspired systems, bringing together neural networks and cellular automata. NCAs work on an image grid with an extended channel dimension, where a common local rule is applied to each of the cells in an iterative fashion. Figure 2 illustrates the operation that is performed on each cell, in each timestep: First, the Moore neighborhood of each cell is aggregated by applying $3 \times 3$ image filters. We use three filter banks, one consisting of identity filters which only yield the current cell’s state, and two learned filter banks that aggregate the cell’s neighborhoods using a different filter matrix in each channel. The scalar result for each channel is stored in a vector that is passed to an multilayer perceptron (MLP), representing the common rule applied to every cell. The final result is then added to the original image buffer once all new cell states are computed. However, only 80% of cells are stochastically updated in each timestep to relax the simultaneous cell updates on the entire grid. For a more detailed overview of the NCA architecture and its key ideas, we point the interested reader to the comprehensive "Growing NCA" paper by Mordvintsev et al. [16].

### NCA model architecture and training

We train two models, namely **eNCApsulateS** for segmentation and **eNCApsulateD** for depth estimation. eNCApsulateS operates on an 18 channel image, whereas eNCApsulateD uses 22 channels total. In both models, the first three channels are fixed, as these are the RGB channels necessary to store the image data. In the last channel, the NCA produces the segmentation or depth map output, respectively. All channels in between are hidden channels that the NCA model uses to retain information between individual time steps. The hidden channels and output channel are initialized noise, as we found that this increases the robustness of the training.

eNCApsulateD is trained on a subset of the KID2 [17] dataset, which is passed through Depth Anything Model V2 [18] in order to obtain pseudo-ground truth depth maps. The resulting depth maps are automatically curated, as we could not fully trust the foundation model and hence had to remove image samples for which the generated depth maps appear flat. To determine whether a depth map is flat, we leverage its normalized gradient magnitude and accept it if it exceeds a threshold of 1.1. After applying this strategy, 727 annotated samples remained, which were used to train the depth estimator.

eNCApsulateD is trained with a combination of three losses: mean squared error (MSE), structural similarity (SSIM) loss and an image gradient loss, weighed $\lambda _{MSE} = 1.0$, $\lambda _{SSIM} = 1.0$ and $\lambda _{grad} = 0.1$ respectively. During training, we make use of batch duplication as this has improved training stability in prior work [9]. Minibatches have size 8 (duplicated: 16) and are comprised of cropped capsule endoscopic samples , which are downsampled to $64 \times 64$ patches. By resizing, we can cut down the high VRAM requirements during training.

For the qualitative evaluation of eNCApsulateD, we select a subset of the KvasirCapsule dataset [10] with interesting benchmark images, which we sort into five categories, each of which contains five sample images: blood, bubbles, complex folds, debris and foreign body.

**Fig. 3:**
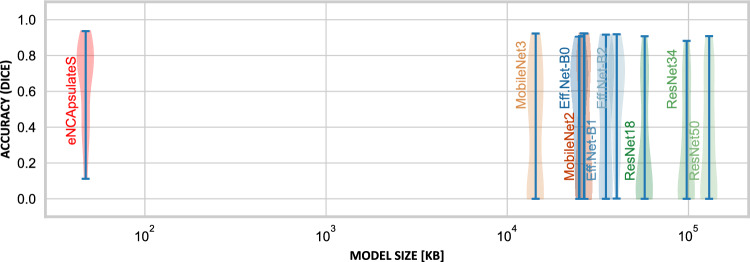
Accuracy of different lightweight segmentation models (blue) vs. eNCApsulateS (green), and their model size in kilobytes, on a logarithmic scale

**Table 1:** Comparison of different small-scale segmentation models (backbones for U-Net) and eNCApsulateS, evaluated on a held-out testset, which is a subset of the KID2 dataset. Results are computed by an ensemble of models trained on the 5-fold-split.

Model	Dice $\uparrow$	IoU $\uparrow$	Size [B] $\downarrow$
nnUNet	0.582	0.462	268,113,106
EfficientNet-B0	$0.465 \pm 0.298$	$0.353 \pm 0.259$	25,005,876
EfficientNet-B1	$0.400 \pm 0.338$	$0.310 \pm 0.289$	35,028,420
EfficientNet-B2	$0.509 \pm 0.288$	$0.389 \pm 0.254$	40,185,164
MobileNetV3s	$0.350 \pm 0.330$	$0.267 \pm 0.276$	14,342,596
MobileNetV2	$0.345 \pm 0.333$	$0.265 \pm 0.285$	26,515,780
ResNet18	$0.251 \pm 0.298$	$0.184 \pm 0.240$	57,312,836
ResNet34	$0.254 \pm 0.291$	$0.184 \pm 0.231$	97,745,476
ResNet50	$0.295 \pm 0.313$	$0.220 \pm 0.257$	130,084,420
eNCApsulateS (Ours)	$0.576 \pm 0.291$	$0.460 \pm 0.270$	**47,152**

**Fig. 4:**
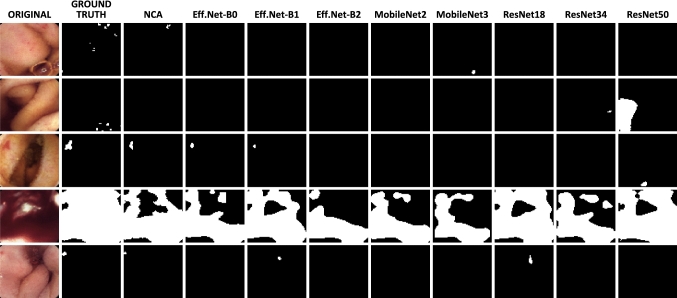
Qualitative segmentation results for eNCApsulateS, compared to other lightweight segmentation models based on CNN

**Fig. 5:**
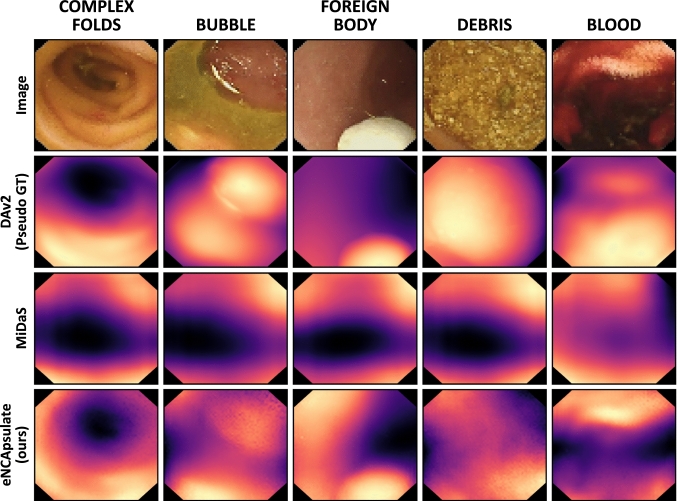
Visual comparison of different monocular depth estimation approaches on a part of the benchmark dataset (subset of KvasirCapsule). eNCApsulate was trained on the KID2 dataset, whereas the other models are foundation models

**Fig. 6:**
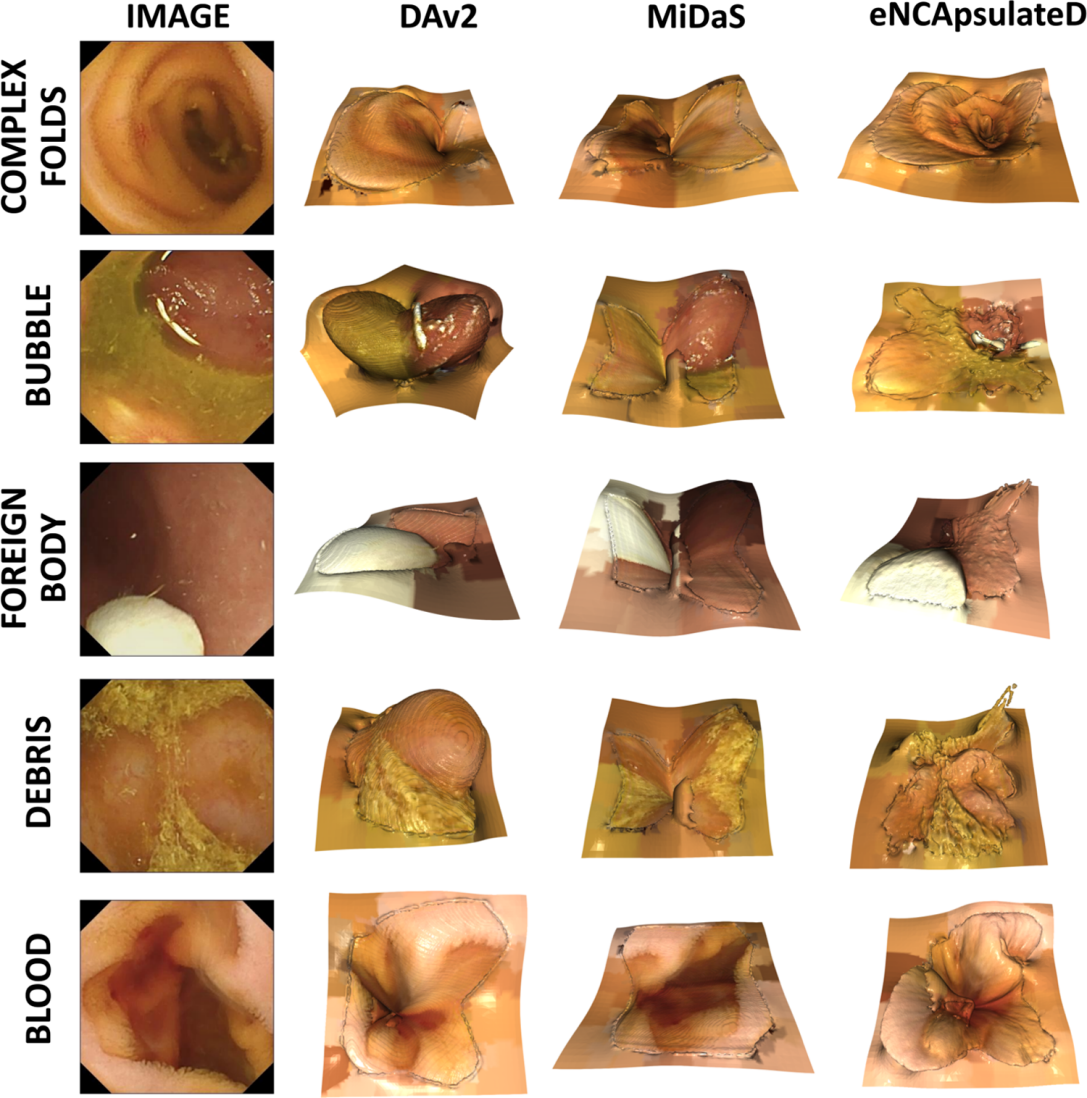
3D Projections of generated RGBD images with baselines and eNCApsulateD, for the five categories of our baseline set

All models are implemented in Python with Pytorch and trained on a PC equipped with an NVidia GeForce GTX 3090.

### Porting NCAs to microcontrollers

Once the NCA model is trained and properly tested on PC hardware, and the next step is to port eNCApsulate to the ESP32-S3 microcontroller. As shown in Figure 1, we use a tiny variant of the ESP32-S3 microcontroller in our experimental setup. Despite not being a dedicated hardware accelerator for neural networks, the ESP32-S3 offers several functionalities that allow us to run NCAs efficiently. Most importantly, it features single instruction multiple data (SIMD) instructions for instruction-level parallelism. These are especially useful for matrix operations, which are needed for the forward pass in the NCA’s MLP. We make use of SIMD instructions whenever applicable, increasing the average runtime speed for a single inference from 9s to 3s. The ESP32-S3 also features a proper floating point unit (FPU), significantly accelerating floating point instructions required for the depthwise convolution.

Since we cannot rely on code optimizations under the hood of a framework like PyTorch, the entire inference loop is implemented from scratch in ANSI C. A major difference in our NCA implementation on the microcontroller is in the order in which the inference steps are executed. The stochastic cell update is the first step to be executed, as it is a 50:50 condition for the rest of the code to run through. After that, we compute the filter operations of the depth-wise convolution; however, we do not store the results of these filters in separate buffer matrices. Instead, we only make use of two buffers: one which is the actual image buffer, and the update buffer which is added to the image after all cell updates were computed. We will provide our implementation upon acceptance of this paper, so that these optimization steps can be traced easily.

### Accelerating inference on the ESP32-S3

Since inference in NCA models is an iterative process, they trade model size for runtime. Although this property allows us to bring the model to lightweight architectures, it comes with the price of a rather slow inference process. Typically, an NCA needs around 100 time steps (forward passes) to converge, which is negligible for inference on the GPU, but costly on the microcontroller, where each time step takes roughly 65ms.

We therefore add an extension to eNCApsulateS in the form of a temporal regularization scheme to reduce unnecessary time steps, in order to reduce the average inference time. In particular, we interrupt the inference process after a certain number of steps if no significant change in the hidden channels is observed. Once a minimum number of steps (10) is reached, we take the total absolute difference between each two consecutive hidden channel tensors, sum them up for all cells and normalize them by the number of entries. If this absolute difference falls below a threshold (we use 0.1), a cooldown counter will be decremented from 5 to 0. Once it reaches 0, the inference is stopped at the current time step, otherwise it is reset to 5. A full description of this algorithm can be found in our public supplemental material.

## Experimental results

**Segmentation with eNCApsulateS:** Our segmentation approach is compared to various lightweight backbones for U-Net, which can also be miniaturized to run on confined hardware architectures. All baselines were pre-trained on ImageNet, with their encoders being frozen before re-training. To estimate the upper bound of what is achievable, we also compare against nnUNet which is the current SOTA for medical image segmentation. All baseline models were trained on the same dataset and five-fold split as eNCApsulateS for comparison. We also used similar settings and hyperparameters for the baseline training (1000 epochs with early stopping, Dice BCE loss, batch size 4) and also used the same image pre-processing. eNCApsulateS clearly outperforms the other lightweight segmentation models that are optimized for size Fig. 3, while being 10x smaller than the smallest baseline (MobileNet V3s) as it is shown in Table 1.

Figure 4 shows that eNCApsulateS performs well on images with different sizes of bleedings, even if they are tiny (bottom row) or excessively large (4th row from above). In some cases, we found that the ground truth labels of KID2 are rather coarse and often oversegment the bleeding which explains the poor upper-bound performance, even for strong models like nnUNet. Even in such cases, eNCApsulateS turned out to be robust as it only delineates the actual bleedings, being nearly on-par with nnUNet.

**Depth Estimation with eNCApsulateD:** NCAs for depth estimation are data efficient and generalize well on an unseen dataset. Reconstructed 3D volumes are demonstrated in Fig. 6. One central limitation of our approach is the absence of a proper ground truth for depth, forcing us to use the best-performing foundation model as a pseudo-ground truth. However, qualitative results (Fig. 5) indicate that eNCApsulateD performs well, and in some cases produces more convincing depth maps than the foundation models. We attribute this phenomenon to two effects: Firstly, the foundation models were trained on a multitude of mostly real-world datasets. Medical data are underrepresented in such datasets, (capsule-)endoscopic data is even less likely to be found. In the end, all models struggle with challenging objects such as bubbles or debris. Secondly, NCA have proven earlier to be robust against data shifts and inconsistencies in training data [9], and they are data efficient thanks to their common local update rule. We assume that they perform well in the segmentation and depth estimation task thanks to these properties, even though the training datasets were rather small.

**Temporal Regularization:** Our temporal regularization approach works well for capsule endoscopic videos, as most frames in capsule endoscopic videos show healthy findings and only few are clinically relevant. In the case of the KvasirCapsule video showing obvious bleedings, our temporal regularization strategy needs 1,222,998 NCA steps total (at the same segmentation quality), whereas 6,988,560 NCA steps are needed without the early cutoff. On the ESP32-S3, this reduction by factor 5 means on average reduction of inference time from 3s to less than 1s per image. As most capsules record frames at 2–3 FPS, such an improvement in runtime speed at similar accuracy implies that accurate bleeding segmentation on the capsule can be performed in reasonable time that aligns with the frequency of recorded images.

## Conclusion

In this paper, we have successfully ported NCAs to the ESP32 microcontroller platform for segmentation and depth estimation on a chip as small as a capsule endoscope. While such tasks typically require large models with sizes of several megabytes, we manage to train models of less than 70 kB that produce convincing segmentations and depth maps. Our segmentation model eNCApsulateS segments the bleedings more accurately than U-Net-based small-scale models, and manages to perform a full image segmentation in less than three seconds. Inference can be further accelerated by stopping the inference process at low hidden channel activity without losing precision. The depth estimator eNCApsulateD predicts depth maps with realistic appearance even in difficult cases (e.g., folds of the colon, dark areas, bubbles), making NCAs a promising candidate technology in confined, but also in less confined settings. However, a hurdle that is yet to overcome is the overall data scarcity and the quality of ground truth in the field of WCE, which can be mitigated by an increase of shared data repositories and advancements of simulation environments like VR-Caps [19]. In future, we plan to integrate the depth estimation approach with similarly lean feature extraction methods to attempt a localization of the capsule camera. To accomplish this goal, absolute depth estimation is necessary, which is already incorporated in models like DepthAnythingV2 and EndoSfMLearner and will be further investigated for eNCApsulateD. Our work paves the way for such a localization strategy, hopefully enabling sensor-less navigation of capsules within the GI tract.

## Supplementary Information

Below is the link to the electronic supplementary material.

Supplementary file 1 (pdf 179 KB)

## Data Availability

KvasirCapsule is available under a public license. KID2 is distributed under a custom academic license by request.

## Code availability

Our code is available on GitHub: https://github.com/MECLabTUDA/eNCApsulate
