# Supplementary material for: eNCApsulate: neural cellular automata for precision diagnosis on capsule endoscopes
Source: Int J Comput Assist Radiol Surg. 2025 Jul 4;21(1):195–202. doi: 10.1007/s11548-025-03425-x (PMC12929257; doi:10.1007/s11548-025-03425-x)
Supplement: Supplementary file 1 — (pdf 179 KB) [file 11548_2025_3425_MOESM1_ESM.pdf]

Supplemental Material for IPCAI'25: eNCApsulate: NCA for Precision Diagnosis on Capsule Endoscopes

Henry Krumb and Anirban Mukhopadhyay, TU Darmstadt

2025

Algorithm 1 Pseudocode for the NCA inference algorithm running on the ESP32-S3, used in both eNCApsulateS and eNCApsulateD. Operations with an underline / yellow highlight are vectorized with SIMD instructions.

```
declare X_input_{channels×3}
declare y_hidden_{hidden_dimension}
declare y_final_{channels}
declare δ_{width,height,channels}
for i = 0 to generations do
    for y = 0 to height do
        for x = 0 to width do
            if random() > 50% then
                continue
            end if {Stochastic cell update}
            let offset ← 0 {Index offset for filter bank results}
            for f = 0 to filters do
                X_input_{offset:offset+channels} ← conv(I_{x,y}, F_f)
                offset ← offset + channels
            end for
            y_hidden ← mat_mul(X_input, W_hidden)
            y_hidden ← y_hidden + B_hidden
            y_hidden ← ReLU(y_hidden)
            y_final ← mat_mul(y_hidden, W_final)
            copy δ_{x,y,0:channels} ← y_final
        end for {x to width}
    end for {y to height}
    for c = 0 to channels do
        for y = 0 to height do
            for x = 0 to width do
                I_{x,y,c} ← I_{x,y,c} + δ_{x,y,c}
            end for {x to width}
        end for {y to height}
    end for {c to channels}
end for {i to generations}
```

```python
def forward(
    self,
    x,
    steps: int = 1,
    auto_step=False,
    auto_max_steps=100,
    auto_min_steps=10,
    auto_plateau=5,
    auto_verbose=False,
    auto_threshold=1e-2,
    return_steps=False,
):
    if auto_step:
        # Assumption: min_steps >= 1; otherwise we cannot compute distance
        assert auto_min_steps >= 1
        assert auto_plateau >= 1
        assert auto_max_steps > auto_min_steps
        cooldown = 0
        for step in range(auto_max_steps):
            with torch.no_grad():
                if step >= auto_min_steps:
                    # normalized absolute difference between two hidden states
                    score = (hidden_i - hidden_i_1).abs().sum() / (
                        hidden_i.shape[0]
                        * hidden_i.shape[1]
                        * hidden_i.shape[2]
                        * hidden_i.shape[3]
                    )
                    if score >= auto_threshold:
                        cooldown = 0
                    else:
                        cooldown += 1
                    if cooldown >= auto_plateau:
                        if auto_verbose:
                            print(f"Breaking after {step} steps.")
                        if return_steps:
                            return x, step
                        return x
            # save previous hidden state
            hidden_i_1 = x[
                ...,
                self.num_image_channels : self.num_image_channels
                + self.num_hidden_channels,
            ]
            # single inference time step
            x = self.update(x)
            # set current hidden state
            hidden_i = x[
                ...,
                self.num_image_channels : self.num_image_channels
                + self.num_hidden_channels,
            ]
        if return_steps:
            return x, auto_max_steps
        return x
```

Figure 1: Algorithm for accelerating inference on time sequence data.
